# Brown Algae and Basalt Meal in Maintaining the Activity of Arylsulfatase of Soil Polluted with Cadmium

**DOI:** 10.1007/s11270-017-3449-7

**Published:** 2017-07-07

**Authors:** Magdalena Zaborowska, Jan Kucharski, Jadwiga Wyszkowska

**Affiliations:** Department of Microbiology, University of Warmia and Mazury in Olsztyn, Plac Łódzki 3, 10-727 Olsztyn, Poland

**Keywords:** Cadmium, Arylsulfatase, *Pseudomonas* sp., Algae, Basalt meal, Soil

## Abstract

This study analysed the effectiveness of innovative (basalt meal, brown algae extract) and conventional (barley straw) substances which hypothetically alleviate the inhibiting effect of Cd^2+^ on biochemical properties of soil, with particular regard to the activity of arylsulfatase. An analysis of their potential was carried out based on the activity of arylsulfatase and the number of *Pseudomonas* sp. determined on the 25th and 50th days of the study. Cd^2+^ was applied in the following doses: 0, 4, 40, 80, 120, 160, 200 mg Cd^2+^ kg^−1^ of DM soil, in the form of CdCl_2_·2.5H_2_O. A complex formulation of the issue was obtained from the presentation of biochemical properties using the RS (resistance of soil) index. Cadmium caused permanent adverse effects in the soil environment, inhibiting the activity of arylsulfatase and the yield of spring barley. The consequences of stress connected with increasing Cd^2+^ pollution were intensified by an elongation of the accumulation time of the tested metal in the soil. Chances for regeneration of the soil may be sought, most of all, with the application of straw and, to a lesser degree, with basalt meal. Brown algae did not meet the expectations for its potential. An increase in the studied parameters also resulted from sowing the soil with spring barley.

## Introduction

Rapid industrialisation and urbanisation and the resulting high global demand for Cd^2+^, have raised concerns about the effects of environmental pollution with cadmium and other heavy metals. Long-term exposure to cadmium is associated with kidney damage, bone mineral loss, increased risk of bone fractures and reduced lung function (ASTDR 2012). Phosphatic fertilisers, sediments (Smith 2009), electroplating, sites for the disposal of waste from industrial processes (Khan et al. 2008) and the manufacture of plastics and paint pigments (Rao et al. 2010) are sources of Cd^2+^ in the soil. It is estimated that over 8 million mg of phosphate fertilizer were used in the USA in 2010 (ASTDR 2012). The EPA ceiling limit for the cadmium content of sludge applied to land is 85 mg kg^−1^ in sewage sludge and the maximum annual cadmium loading is 1.9 kg^−1^ ha^−1^ year^−1^ (EPA 2011). Its availability in the soil environment is affected by various factors, including soil pH (Houben et al. 2013), content of dissolved organic carbon (Longhua et al. 2012) and plant species (Mahmood et al. 2014). Nevertheless, the accumulation of Cd^2+^ in the soil, disturbing its homeostasis, is the most serious problem. Symptoms of the adverse influence of Cd^2+^ on the soil ecosystem may also be found in its biochemical properties (Kucharski et al. 2011; Wyszkowska et al. 2013), which correspond directly with soil fertility and plant yield (Oropeza-Garcia et al. 2014). Some of these symptoms include inhibition of chlorophyll biosynthesis, the activity of δ-aminolaevulin acid synthase and protochlorophyllide reductase (Macfarlane and Burchett 2001). This results in oxidation stress, leading to an increased peroxidation of lipids and the production of a reactive form of oxygen—hydrogen peroxide—in both roots and leaves (Hao et al. 2006). Exposure to high Cd^2+^ doses, exceeding the tolerance of plants, is directly connected with an increase in ethylene level, and thus the activity of ACC (1-aminocyclopropane-1-carboxylic acid) which is responsible for its synthesis (Maksymiec et al. 2007). One method for the decrease of Cd^2+^ availability to plants consists in increasing the amount of phosphorus (Mohamed et al. 2012). Data based on in vitro examination of the genotoxic effects of cadmium in microorganisms, suggests that this xenobiotic has the potential to induce DNA damage, micronuclei, chromosomal aberrations and genetic mutations (ASTDR 2012).

Among the numerous reliable parameters for evaluation of the soil condition, enzymatic activity is among those most often used (Jezierska-Tyś and Frąc 2006). Arylsulfatase—apart from dehydrogenases, urease and phosphatases—is most frequently used as an indicator for the pollution of soils with heavy metals and pesticides (Gil-Sotres et al. 2005; Zaborowska et al. 2016). There is also a growing need for the quantification of the activity of enzymes, both intra- and extracellular, to which arylsulfatase belongs, in order to create an opportunity for a reliable analysis of the microorganism potential in soil transformations (Zwikel et al. 2007). Arylsulfatase (sulfohydrolase, EC 3.1.6.1) is an important enzyme because of the common occurrence of sulphur in the soil and making it available to plants in an oxidation process. This enzyme hydrolyses aromatic sulphate (VI) esters (R-O-SO_3_^2−^) to phenols (R-OH) and inorganic sulphates (VI) (SO_4_^2−^) (Elsgaard and Vinther 2004). In most soils, 95% of the sulphur content is represented by sulfonates and sulphate esters. According to Kahnert and Kertesz (2000), *Pseudomonas putida* has regulatory proteins (AsfR) which are necessary for the desulphonation of aromatic and aliphatic sulfonates. Directed evolution, generated by an intention to improve the enzymatic properties of soils, contributed to the isolation of an H260L arylsulfatase from a *Pseudoalteromonas carrageenovora* mutant, which is an enzyme much more resistant to the variable properties of the solum (Zhu et al. 2017). Arylsulfatase activity is closely correlated with the microbial activity (Vong et al. 2010). Kertesz et al. (1993) demonstrated that the reduction in the source of sulphates resulted in the specific expression of proteins in *Pseudomonas* sp., contributing to an increase in arylsulfatase activity by up to 140 times. The proteins induced under these conditions were involved in sulphur metabolism. They are referred to as sulphate starvation-induced (SSI) proteins and have been divided into three groups based on the functions they serve in response to sulphur deficit. This is why the enzyme is frequently considered to be the SSI protein of groups I and III, synthesized by microorganisms (Byun et al. 2004), mainly characterized by *Protobacteria* (Berteau et al. 2006) and fungi *Eupenicillium* sp. or *Trichoderma* sp. (Slezack-Deschaumes et al. 2012). The discovery of the regulation of arylsulfatase activity at the transcriptional level allows one to conclude that arylsulfatase synthesis may also be induced to release sulphates from alternative sulphur sources such as sulphuric acid esters (Piutti et al. 2015).

The effects of the application of mitigating substances depend on a multiplicity of transformations occurring in the soil. Eventually, fertilisation may neutralise the consequences of the pressure that the soil polluted with Cd^2+^ has been subjected to. The use of algae for this purpose may prove a good solution, in spite of the fact that various preferences of algae towards the individual metals (Cu > Cd > Zn) have been found (Yoshida et al. 2006). However, their adsorptive properties connected with the use of a proper biomass of bacteria make them attractive biosorbents (Munoz et al. 2006), in the presence of organic compounds (Abinandan and Shanthakumar 2015). However, it should be noted that the regenerative potential of algae may be highly strain-specific. Monteiro et al. (2010) noted a 25% difference in the biosorption of Cd^2+^ by two strains of *Desmodesmus pleiomorphus.* Straw, as an unrivalled source of carbon, is also a good material for fertilisation of the soil environment (Badía et al. 2013). Basalt meal, with the co-participation of an additional carbon source, is also able to increase the plant yield (Anda et al. 2013). According to Nunes et al. (2014), basalt from mining waste can be used as a fertiliser, thus reducing the scope of the disposal of such waste while contributing to sustainable development.

In order to reliably evaluate the chances for maintaining or restoring the equilibrium in the soil subjected to strong Cd^2+^ pressure, changes in its stability over time were traced, by determination of the indicators of the resistance of soil (RS) to arylsulfatase. The analysed parameter was also presented as a rhizosphere effect (R:S), taking into account the influence of spring barley. Moreover, the evaluation of the influence of Cd^2+^ on the yield of the cultivated plant was carried out. However, the primary goal was to compare the efficiency of the alleviation of adverse effects of Cd^2+^ accumulation in the soil by basalt meal and brown algae extract in relation to the conventional method of fertilisation with barley straw.

## Material and Methods

### Soil and Experimental Procedure

Soil used in the vegetation experiment originated from the Didactic and Research Centre in Tomaszkowo situated in NE Poland (53.71610 N, 20.41670 E). The zone for the research has a surface area—including protective belts—of approximately 4.5 ha. The soil material was sampled from the topsoil of typical brown earths (Eutric Cambisol). According to the graining classification of the United States Department of Agriculture, it is a soil with a granulometric composition of loamy sand. The soil physicochemical characterisation has been performed (Table 1). The following soil properties were defined: reaction (pH) by potentiometric in an aqueous solution of KCl of the concentration of 1 mol dm^3^ (ISO 10390, 2005); hydrolytic acidity (HAC) and exchangeable base cations (EBC)—by the Kappen method (Klute 1996), content of organic carbon (Corg)—by the Tiurin method (Nelson and Sommers 1996). Based on the HAC and EBC values, the cation exchange capacity (CEC) and base saturation (BS) of the soil were computed. The following equations were applied: CEC = EBC + HAC; BS = (EBC/CEC)·100.

**Table 1:** Some physicochemical properties of soil used in the experiment

Properties	Unit	Value
Granulometric composition of soil (percentage of fraction (*d*))	2.00 ≥ *d* ≥ 0.05 mm	75
	0.05 ≥ *d* > 0.002 mm	20
	*d* ≤ 0.002 mm	5
pH_KCl_		5.8
HAC	mM (+) kg^−1^ DM of soil	14.75
EBC		48.67
CEC		63.42
BS	(%)	76.75
Corg	g kg^−1^ DM of soil	6.4

*HAC* hydrolytic acidity, *EBC* sum of exchangeable cations, *CEC* cation exchange capacity, *BS* base saturation, *pH*_*KCl*_ soil reaction

The second stage of the studies was realized in the plant house of the University of Warmia and Mazury in Olsztyn (NE Poland), based on a pot experiment carried out in five replications.

The influence of the following variables was evaluated: (1) degree of pollution of the soil with cadmium in mg Cd^2+^ kg^−1^ DM of soil: 0, 4, 40, 80, 120, 160, 200, (2) addition of fertilizing substances: basalt meal, Labimar 10S—algae extract, finely ground straw of spring barley, (3) soil use: unsown treatments and treatments sown with spring barley (*Hordeum vulgare* L.) and (4) duration of the studies: 25, 50 days.

Before commencing the experiment, the soil material was prepared by polluting it with cadmium (CdCl_2_), adding NPKMg fertilisers, and potentially alleviating substances (in proper subjects). After mixing the soil in a polyethylene pot, it was packed into pots (3.5 dm^3^), in the amount of 3.2 kg per pot. Subsequently, the level of humidity was modulated to 60% of capillary water capacity in all objects. One level of fertilization with macro- and microelements was applied, which were converted to a pure component in mg kg^−1^ of soil: N – 250 [CO(NH_2_)_2_], P – 50 (KH_2_PO_4_), K – 90 (KH_2_PO_4_), Mg – 20 (MgSO_4_. 7H_2_O), Cu – 5 (CuSO_4_. 5H_2_O), Zn – 5 (ZnCl_2_), Mo – 5 (NaMoO_4_. 2H_2_O), Mn – 5 (MnCl_2_. 4H_2_O) and B – 0.33 (H_3_BO_3_) were used in all experiments. Spring barley of the *Rabel* cultivar was then sown at certain sites. After sprouting, plants were segregated and 15 items were left per pot. The plant vegetation period was 50 days. The dry matter yield was determined after harvesting the spring barley (BBCH 52, 20% of inflorescence emerged).

### Substances Used for Biostimulation

Basalt meal (Stomeb PPHU, Mietków, Poland) and brown algae extract—Labimar 10S (P.U.H. “Polger-Kido”, Słupsk, Poland)—were experimentally applied to the soil in doses 0 and 5 g kg^−1^ DM of soil (basalt meal) and 0 and 1.56 cm^3^ kg^−1^ DM of soil (Labimar 10S), respectively. Basalt meal includes: P – 1.7, K – 10, Ca – 68.5, Mg – 40.9, Si – 231.4, Al – 84.7, Fe – 93.5, Na – 21.5, Mn – 1.5 (g kg^−1^) and trace amounts of Zn, Cu, B, Mo, Co. The extract of brown algae included: dry matter (45%), organic matter (36% – amino acids, vitamins, mono-, poly- and oligosaccharides, enzymes, phytohormones) and organic boron (2.5%). The content of elements for these substances was determined by producers. Finely ground barley straw was introduced in doses 0 and 5 g kg^−1^ DM soil, as an alternative conventional method for the reclamation of soil. All substances were introduced into the soil. The impact of substances was determined based on the impact coefficient of an alleviating substance that was calculated with the following formula (Zaborowska et al. 2015):

$$ \mathrm{IF}=\frac{\mathrm{Ss}}{\mathrm{Sc}} $$

where:

IF: coefficients of fertilization effect of an alleviating substance (IF <1—an alleviating substance does not positively impact arylsulfatase activity or the number of *Pseudomonas* sp., IF >1—an alleviating substance stimulates the analysed soil parameters),
Ss: activity of arylsulfatase or the number of *Pseudomonas* sp. in soil with an alleviating substance,
Sc: activity of arylsulfatase or the number of *Pseudomonas* sp. in soil without an alleviating substance.

### Determination of the Activity of Soil Arylsulfatase and the Number of *Pseudomonas* sp.

The soil samples collected on day 25 and 50 of the experiment were tested for arylsulfatase activity (EC.3.1.6.1) with the method described by Alef and Nannipieri (1998). Potassium - 4 nitrophenylsulphate (PNS) was the substrate used to determine this enzyme. From each soil sample, 1 g of the soil, 4 cm^3^ 0.5 M acetate buffer at pH = 5.8 and 1 cm^3^ 0.02 M solution of PNS were added to it successively. The samples were incubated for 1 h at 37 °C. After the incubation, 25 cm^3^ distilled water was added to every sample. The content of each sample was then thoroughly mixed. After filtering, 4 cm^3^ 0.5 M NaOH was added to 6 cm^3^ of filtrate. This operation was repeated three times. Extinction of the produced 4 - nitrophenol was measured on an Aquarius CE7500 (Cecil Instruments) spectrophotometer, at a wavelength of *λ* = 420 nm. The activity of arylsulfatase was expressed in micromoles PNP kg^−1^ DM of soil per hour. The soil samples were tested for the number of *Pseudomonas* sp. on a medium described by Wyszkowska et al. (2008). The number of microorganisms was determined with a colony counter. The recorded results are depicted as the rhizosphere effect (R:S), i.e. the ratio of arylsulfatase activity and number of *Pseudomonas* sp. in soil sown with spring barley (R) to the same parameters in unsown soil (S). The activity of arylsulfatase and spring barley yielding were used to evaluate the resistance of soil (RS) to contamination with cadmium. Calculations were made with the formula proposed by Orwin and Wardle (2004).

$$ \mathrm{RS}=1-\frac{2\ \left|\  D0\left.\right|\right.}{C0 + \left| D0\left.\right|\right.} $$

where: *D*_0_—the difference between control soil (*C*_0_) and contaminated soil after 25 days of incubation (*t*_0_). The control soil (*C*_0_) is not contaminated with cadmium and not biostimulated by any of the proposed substances. The values of RS remain in the range of 0 to 1, where 1 indicates strong soil resistance, i.e. negligible effects of external factors. The lower the resistance, the stronger the impact of a given factor on the soil environment.

### Statistical Analysis

The results were statistically processed using the statistica v. 12.0 (Statsoft, Inc., Statistica 2016) statistical software package. Homogenous groups were calculated with Tukey’s test at *P* = 0.01. The coefficients of a simple Pearson correlation test of the increasing cadmium doses and the activity of arylsulfatase were determined. The rhizosphere effect after 25 and 50 days was illustrated using principal component analysis (PCA). The biostimulation *Pseudomonas* sp. counts on fertilizing substances was determined by cluster analysis and presented in a dendrogram in accordance with Ward’s method. The percentage of variation of all investigated variables (*η* ^2^) was determined with an analysis of variance (ANOVA). The following formula was used in this method:

$$ {\eta}^2=\frac{{\mathrm{SS}}_{\mathrm{effect}}}{{\mathrm{SS}}_{\mathrm{total}}}\bullet 100\% $$

where:

*η*^2^: coefficient *η* ^2^
SS_effect_: sum of squares for the analysed effect,
SS_total_: sum of squares for all effects.

## Results and Discussion

### Effect of Soil Contamination with Cadmium on Arylsulfatase Activity

**Fig. 1:**
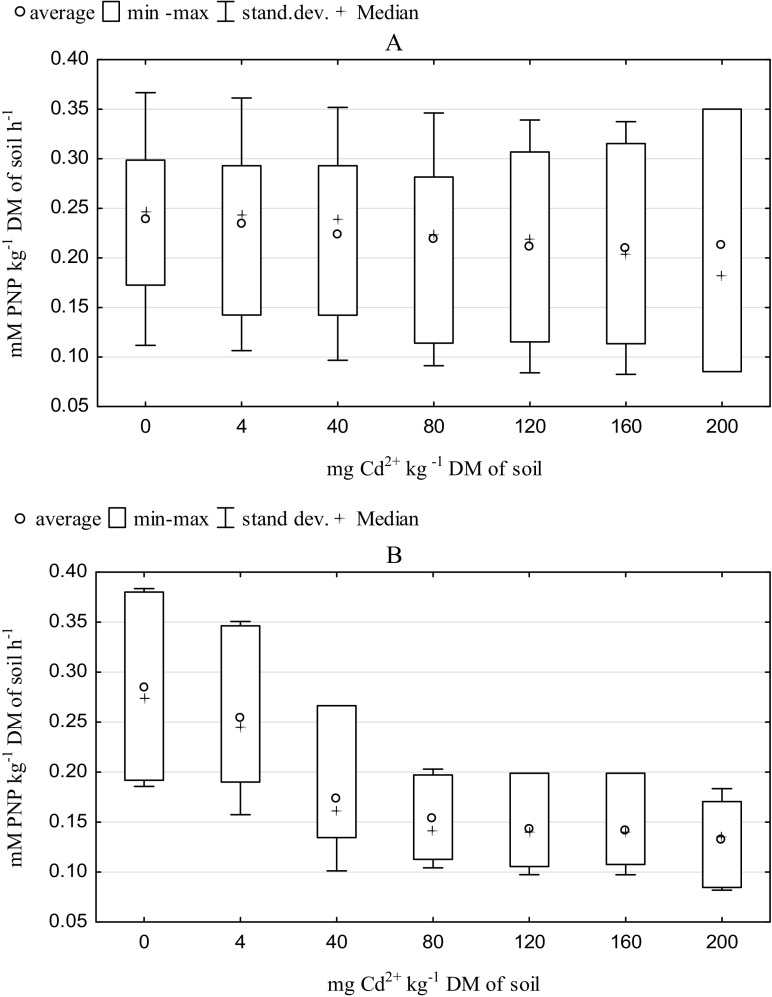
Effect of soil contamination cadmium on arylsulfatase activity in the unsown (**a**) and sown soil (**b**), mM PNP kg^−1^ DM soil h^−1^

The results of these studies concerning the influence of Cd^2+^ on the biochemical activity of the soil correspond to the results obtained by other authors (Mikanova 2006; Zaborowska et al. 2015). Analysis of the η2 coefficient proved that the dose used (24%), the accumulation time of cadmium in the soil (14%), and the soil use (8%) significantly modified the activity of arylsulfatase. In the subjects sown with spring barley, doses in the range of 40–200 mg Cd^2+^ kg^−1^ DM of soil caused a stronger adverse influence on the activity of arylsulfatase than in the soil samples not used for cultivation (Fig. 1a, b). Knauff et al. (2003) observed the highest activity of arylsulfatase at a distance of 1 mm from the root. However, Renella et al. (2006) noted an inhibition of the synthesis of citric acid, glutamic acid, oxalic acid and glucose in soil polluted with 20 mg Cd^2+^ kg^−1^ DM of soil. ^13^C and ^14^C studies showed very high initial rate of glucose mineralization (1.1% min^−1^) and much higher rate of sugars uptake by microorganisms from the soil solution (Gunina and Kuzyakov 2015). Researchers speculate that the most important functions of sugars in soil are to maintain and stimulate microbial activities in the rhizosphere leading to mobilization of nutrients by accelerated SOM decomposition. There is a strong correlation between microbial biomass-S and arylsulfatase activity (Vong et al. 2003). Therefore, cadmium pollution of soil already at dose of 40 mg kg^−1^ DM of soil can causes strong stress reaction of microorganisms related to reduced availability of organic matter. It also indicates higher arylsulfatase activity in cadmium contaminated samples at doses of 0 and 4 mg Cd kg^−1^ DM of soil in sown soil. Then, the strategy of their survival was induced, which caused their biodiversity to change. For example, for *Microbacterium* and *Rhodococcus* isolates, only a membrane arylsulfatase was found. The membrane arylsulfatase was induced both by substrate presence or S demand independently (Cregut et al. 2013). Potentially, the activity of these microorganisms could be inhibited.

**Fig. 2:**
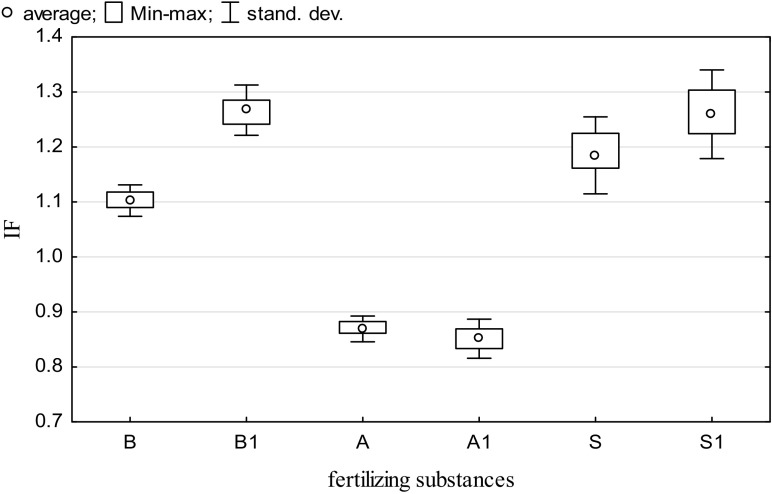
The influence of fertilizing substances (IF) on arylsulfatase activity in the unsown (B, A, S) and sown (B1, A1, S1) soil; *B* basalt meal, *A* algae, *S* barley straw

The application of fertilizing substances did not give the expected effects in alleviating the results of Cd^2+^ inhibiting impact (Fig. 2). However, both in the pool of subjects sown and unsown with spring barley, a slight increase in the activity of arylsulfatase after the introduction of basalt meal and straw to the soil was observed. Brown algae extract did not meet the expectations of its potential.

The toxicity of Cd^2+^ for the individual enzymes, including arylsulfatase, was also proven by Mikanova (2006). This enzyme proved to be more sensitive to the tested metal than urease and invertase, and the adverse influence on its activity was observed after the application of just 2.4 mg Cd^2+^ kg^−1^ DM of soil. Dar (1996) observed an inverse dependence in the presence of 10 mg Cd^2+^ kg^−1^ DM of soil and the toxic properties of the tested metal became evident only with the dose of 50 mg Cd^2+^ kg^−1^ DM of soil. The sensitivity of arylsulfatase to heavy metals close to that of other hydrolases, such as acid phosphatase and alkaline phosphatase, was also noted by Wyszkowska et al. (2010).

The use of indicators providing a chance for the complex analysis of the fertility of the soil under increasing pressure from Cd^2+^ contributed to an increase in the reliability of its stability evaluation. Most of all, however, it emphasised the interdependence between extending the accumulation time of this metal in the soil and its inhibiting power. The resistance (RS) of arylsulfatase was decreased from 36 to 50% (Table 2) compared to the samples with 4 mg Cd^2+^ kg^−1^ DM of soil, on the 25th and 50th incubation days, respectively.

**Table 2:** Indicators of arylsulfatase resistance (RS) to soil contamination with Cd^2+^, subject to the applied soil fertilizing substances after 25 and 50 days

Dose Cd (mg kg^−1^ DM of soil)	Control		Basalt meal		Algae		Barley straw	
	25	50	25	50	25	50	25	50
Unsown								
4	0.876^a^	0.856^a^	0.750^a^	0.865^a^	0.887^a^	0.271^bcd^	0.910^a^	0.466^bc^
40	0.778^ab^	0.834^a^	0.597^ab^	0.933^a^	0.700^abc^	0.283^bc^	0.798^ab^	0.557^abc^
80	0.725^ab^	0.768^a^	0.431^bc^	0.881^a^	0.633^bcd^	0.225^bcd^	0.794^ab^	0.598^abc^
120	0.660^abc^	0.750^a^	0.344^c^	0.864^a^	0.501^cde^	0.199^bcd^	0.725^abc^	0.598^abc^
160	0.546^bcd^	0.642^ab^	0.344^c^	0.747^ab^	0.490^cdef^	0.131^de^	0.637^abc^	0.855^a^
200	0.558^bcd^	0.429^bcd^	0.337^c^	0.747^ab^	0.382^ef^	0.015^e^	0.563^bcd^	0.826^ab^
Average	0.691	0.713	0.468	0.839	0.599	0.187	0.738	0.643
*r*	−0.976*	−0.925*	−0.907*	−0.829*	−0.970*	−0.940*	−0.982*	0.935*
Sown								
4	0.909^a^	0.591^abc^	0.767^a^	0.838^a^	0.863^ab^	0.629^a^	0.879^a^	0.963^a^
40	0.529^bcd^	0.374^cd^	0.603^ab^	0.483^bc^	0.421^def^	0.558^a^	0.509^cde^	0.574^abc^
80	0.529^bcd^	0.266^d^	0.582^ab^	0.465^cd^	0.287^ef^	0.397^ab^	0.300^de^	0.317^c^
120	0.455^cd^	0.250^d^	0.542^b^	0.369^cd^	0.255^ef^	0.359^abc^	0.278^e^	0.317^c^
160	0.418^cd^	0.257^d^	0.542^b^	0.336^d^	0.264^ef^	0.372^abc^	0.285^e^	0.297^c^
200	0.347^d^	0.250^d^	0.502^bc^	0.336^d^	0.243^ef^	0.283^abc^	0.281^e^	0.270^c^
Average	0.531	0.313	0.590	0.471	0.389	0.425	0.422	0.421
*r*	−0.861*	−0.673*	−0.875*	−0.846*	−0.788*	−0.853*	−0.807*	−0.722*

Same letters for a given fertilizing substances in columns are assigned to homogenous groups

*r* correlation coefficient

*Significant for *P* = 0.01, *n* = 17

The particular alleviating properties of basalt meal, which became evident after 50 days of incubation of the soil, and those of barley straw after 25 days of the experiment, are noteworthy. Basalt meal dissolves very slowly in nature, therefore, its stimulating effects are manifested after a long period of time (Shamshuddin et al. 2011). However, according (Shazana et al. 2013) under acid sulphate soil conditions (pH < 3.5), basalt is expected to dissolve much faster.

This was also demonstrated by the formed groups with homogenous variances. In the soil samples sown with spring barley, comparable dependences were found. However, cadmium generated a lower resistance of arylsulfatase, both on the 25th and 50th days of the experiment. In this group of subjects, it decreased by 62 and 57%, respectively, in relation to the samples with 4 mg Cd^2+^ kg^−1^ DM of soil applied. However, it was observed that none of the mitigating substances was able to improve the biochemical properties of the soil to a degree enabling reaching of a state Table 2 of equilibrium after the stress connected with pollution of the soil with the tested metal (Table 2). However, both brown algae extract and straw improved the resistance of arylsulfatase in the presence of 4 mg Cd^2+^ kg^−1^ DM of soil, particularly effectively on the 50th day of the experiment. These substances increased the RS values by 158 and 106%, respectively.

Undoubtedly, an excess of the retained cadmium in the soil leads to prolonged adverse effects for the biochemical properties of the soil. Lorenz et al. (2006) states that after 25 years, soil polluted with this metal in the amount of 50 and 250 mg Cd^2+^ kg^−1^ DM of soil still contained 34 and 134 mg Cd^2+^ kg^−1^ DM of soil, respectively. An effective method for abatement of its toxic effect consists in using barley straw which, in studies by Wyszkowska et al. (2013), generated significantly higher values of resistance of the individual enzymes. The application of straw to soil has received great attention because of its potential benefits such as fertility improvement and carbon (C) sequestration. Straw amendment significantly increased respiration rate, total phospholipid fatty acids (PLFAs) and ^13^C-PLFA especially actinomycetes, Gram-positive bacteria and fungi (Pan et al. 2016). Microbial diversity and their populations are the important factors that govern the enzyme activities in soil. In turn, the enzyme activity in soil acts as the sensor of soil microbial and nutrient status and its fertility (Jain and Pandey 2016). However, the increasing CO_2_ emission as a result of its application cannot be omitted (Badía et al. 2013). One may also suppose that basalt meal will perform the expected alleviating role on the 50th day of the research because of a slow rate of dissolution under natural conditions (Shamshuddin et al. 2011). Its effectiveness is also supported by the release of Ca, Mg, K and Na cations in the form of both exchangeable and soluble cations. The magnitude of exchangeable cations, in order of decreasing content, was Mg > Ca > K > Na, while the order of soluble cations was Na > Mg > Ca > K (Anda et al. 2015). What was surprising was the lack of significant biostimulation in the presence of brown algae. It should be emphasised that against the background of other algae, brown algae are characterised by exceptional sorptive properties for heavy metals (Romera et al. 2007). This is due to the occurrence of an alginate characterised by high affinity for biosorption in their cell wall. It should be stressed, however, that the process is stimulated by numerous factors: pH, temperature, algae biomass, metal ion concentration and the presence of competitive ions (Zeraatkar et al. 2016).

### Effect of Soil Contamination with Cadmium on the Values of Rhizosphere Effect R:S

**Fig. 3:**
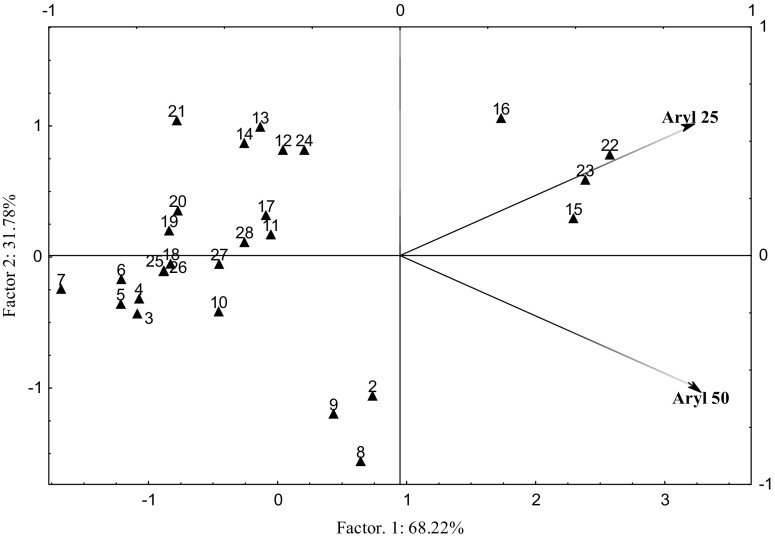
The rhizosphere effect (R:S) arylsulfatase activity (average values): in soil contaminated with Cd^2+^ after 25 and 50 days. Dose Cd^2+^, mg kg^−1^ DM of soil: 0 (cases: 1, 8, 15, 22), 4 (2, 9, 16, 23), 40 (3, 10, 17, 24), 80 (4, 11, 18, 25), 120 (5, 12, 19, 26), 200 (7, 14, 21, 28), cases: 1–7 - control, 8–14 with basalt meal, 15–21 with algae, 22–28 with barley straw

The influence of the plant on the condition of soil subjected to pressure from Cd^2+^ was expressed as values of the rhizosphere effect R:S. This dependence was interpreted using the PCA method (Fig. 3).

On the 25th and 50th days of the research, the distribution of vectors around the axis representing the first factor described 68.22% of the total data variance. This means that the activity of arylsulfatase was positively correlated with this variable, irrespective of the time of Cd^2+^ accumulation in the soil. One homogeneous group formed, with positive values of vectors representing the primary component variables. The distribution of cases defined by both PCA axes corresponds to the influence of the used fertilizing substances on the values of the rhizosphere effect. The conducted analysis reflected a lack of any significant positive influence of the innovative substance applied to the soil. Only straw slightly abated the effects of Cd^2+^ inhibition, present in the dose of 40 mg Cd^2+^ kg^−1^ DM of soil. Both on the 25th and 50th days of the research, a comparable efficiency of the applied substances was observed.

**Fig. 4:**
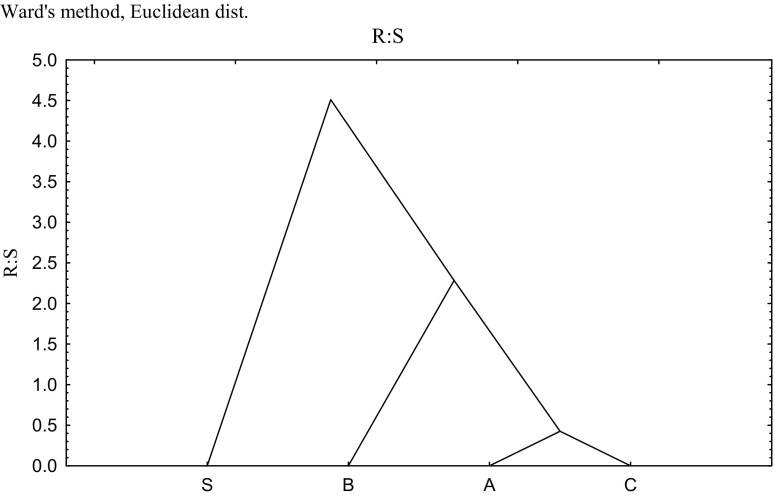
The rhizosphere effect (R:S ratio) for *Pseudomonas* sp. counts in soil samples from control treatments exposed to biostimulation on fertilizing substances in soil contaminated with Cd^2+^. *C* control, *B* basalt meal, *A* algae, *S* barley straw

**Fig. 5:**
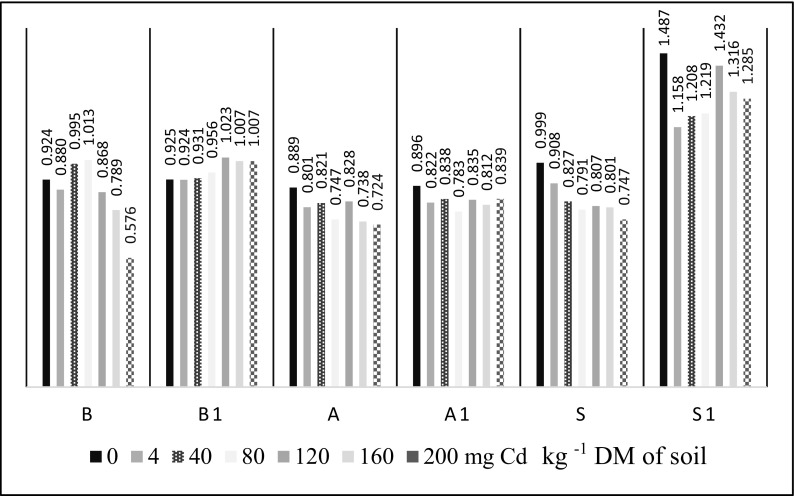
The influence of fertilizing substances (IF) on the number of *Pseudomonas* sp. in the unsown (B, A, S) and sown (B1, A1, S1) soil contaminated with Cd^2+^. *B* basalt meal, A algae, *S* barley straw

The higher biological activity within the rhizosphere zone is well-known and is attributed to the beneficial role of root secretions (Wyszkowska et al. 2009). However, protons H^+^ liberated from the roots may lead to a decrease in pH, thus lowering the enzyme activity. A compensating function is then performed by the higher metabolic activity of the biomass of microorganisms (Knauff et al. 2003). This is confirmed by the results of the authors’ own study (Fig. 4) in which the number of *Pseudomonas* sp. was significantly higher in objects sown with spring barley. In addition, the separate clusters formed by samples fertilized with straw and basalt meal in the cluster analysis by Ward’s method emphasized their significant, beneficial effect on the R:S values for *Pseudomonas* sp. No stimulation was noted for samples with the addition of algae which formed, along with control samples, a group with homogeneous variances. It is the sowing of the soil with the test crop in combination with the fertilization with straw that had the most beneficial effect on the number of *Pseudomonas* sp. (Fig. 5). However, irrespective of the pool of analysed object, the strength of inhibition of the increasing doses of Cd^2+^ contributed to the inhibition of the microbial activity corresponding to arylsulfatase activity.

In a study carried out by Zaborowska et al. (2015), Cd^2+^ had a more adverse effect on the number of cellulolytic and copiotrophic bacteria than it had on the number of *Pseudomonas* sp. In turn, straw generated higher R:S values for *Pseudomonas* sp. than it did for cellulolytic bacteria. An interesting relationship was also discovered by Ayano et al. (2015), who proved that for *Pseudomonas aeruginosa* stain RB, high resistance to cadmium is associated with the biosynthesis of CdSe (cadmium selenide) by this microorganism in the presence of lactic acid as the carbon source. Furthermore, Kilic and Donmez (2008) emphasize that *Pseudomonas* sp. has a certain type of resistance to heavy metals, related to the mechanisms of biosorption or bioaccumulation of metals by an exopolysaccharide (EPS) produced by it. Undoubtedly, *Pseudomonas* sp., besides *Actinobacteria*, moderates arylsulfatase activity in the rhizosphere zone of spring barley (Cregut et al. 2009), and is also a significant microorganism for plants. Singh et al. (2013) emphasize the defensive function of the PHU094 strain of *Pseudomonas aeruginosa* in a plant’s response to biotic stress by activating the induced systemic resistance (ISR) in the host. In turn, the symbiosis of plants with microorganisms as a phytoremediation factor, results in an improvement to the biological activity of soils (Wenhao et al. 2013).

Arylsulfatase has an indirect significant influence on crop quality. Thanks to its common occurrence in the soil, it contributes to making sulphur available to plants (Elsgaard and Vinther 2004). Slezack-Deschaumes et al. (2012) also suggest that microorganisms synthesize arylsulfatase at various stages of the plant’s growth. The following *Actinobacteria* genera were identified: *Arthrobacter*, *Oerskovia* and *Microbacterium*, which are active at each stage of its development. In turn, *Proteobacteria* were associated with *Pseudomonas*, *Klebsiella Raoultella* and *Sinorhizobium*, and revealed their potential principally during the development of seeds. It should be stressed that approx. 41.5% of isolates were associated with *Pseudomonas* sp.

**Fig. 6:**
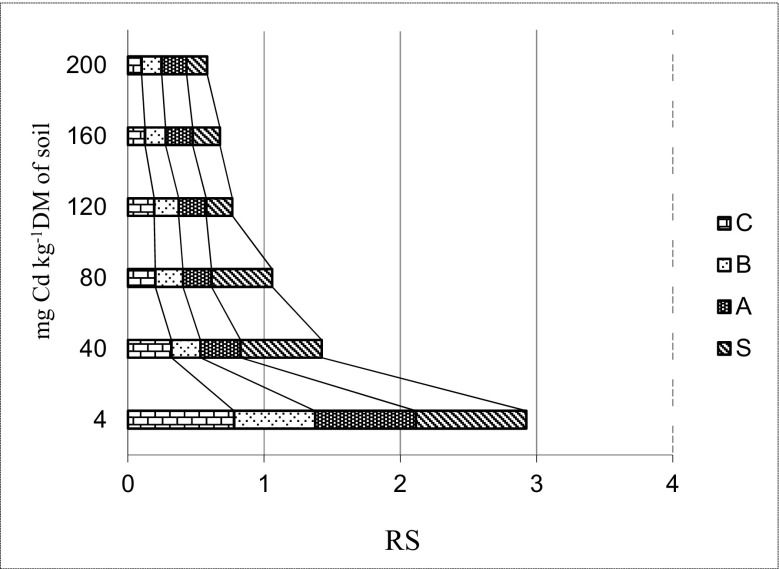
Index of resistance (RS) of spring barley depending on cadmium pollution. *C* control, *B* basalt meal, *A* algae, *S* barley straw

Cd^2+^ introduced to the soil also contributed to a decrease in the yield of the cultivated plant (Fig. 6). Inhibition of growth, chlorosis of leaves and deformation of the root system are some of the observed symptoms of the disturbance of biological mechanisms of spring barley as a result of stress connected with the application of the tested metal to the soil. However, this plant proved to be quite resistant to increasing pollution with Cd^2+^, particularly in doses above 80 mg Cd^2+^ kg^−1^ DM of soil.

With monocotyledonous plants, deficiencies of this element do not become as visually evident as the consequence of excessive Cd^2+^ concentration in the soil. In studies by Wyszkowski and Wyszkowska (2009), the application of 60 mg Cd^2+^ kg^−1^ DM of soil reduced the spring barley crop by half, as a result of photosynthesis disturbance by Cd^2+^, according to Mohamed et al. (2012). The most favourable influence on the resistance of spring barley was exerted by straw. In the group of applied alleviating substances, the effectiveness of algae causes a great deal of controversy. However, the reaction of soil is a significant moderator of their activity. According to Yoshida et al. (2006), brown algae may become attractive biosorbents in the medium with pH > 8 or pH < 5. In addition, the process of metal ion biosorption is complex and usually takes place in two phases. The first phase is independent of the cell metabolism, while the second phase is based on the transport of ions to the cell’s vacuole involving metallothionein (MTs) binding proteins (Monteiro et al. 2012). It is noteworthy that the identification of the significance of specific algae strains responsible for the kinetics and the mechanism of biosorption on the surface of their cells (Lee et al. 2016). *Chlamydomunas reinhardtii* has the capacity to rapidly adsorb Cd^2+^ ions and reaches equilibrium for the biosorption within 60 min (Mata et al. 2009). The defensive response of algae cells to metal ions is largely dependent on their resistance to oxidative damage. The excessive toxicity of Cd^2+^ to algae is reflected by the denaturation of protein structures, total chlorophyll loss, a reduced number of chloroplasts and the inhibition of cell growth (Lamaia et al. 2005). Opinions on the effect of temperature on biosorption vary. Khan et al. (2012) believe that the process is stimulated by its growth, while Gupta et al. (2010) observed that the heavy metal ion capture capacity increased with the decrease in the temperature. According to Aksu (2002), the maximum biosorption of Cd^2+^ by the algae occurred at 20 and 45 °C. Basalt meal did not have such a beneficial effect on the yield of barley as straw had. This may be associated with an observation by Anda et al. (2013) that a short-term phenomenon formation of silicic acid (H_4_SiO_4_) from basalt, which is adverse (from the perspective of microorganism functioning) may occur. This is, however, a short-term process, and the increase in pH value of the soil fertilized with basalt meal is attributed to the increase in exchangeable alkaline cations in soil (Anda et al. 2015; Nunes et al. 2014). In addition, in the presence of basalt meal, the content of toxic Al and Mn decreases (Anda et al. 2015). The positive effects of this were noted by Zuba et al. (2011), who demonstrated that the use of basalt significantly reduced the incidence of soft rot and pests compared to the use of chemicals.

## Conclusions

Arylsulfatase is a sensitive indicator for the pollution of soil with Cd^2+^. Time was shown to be an important moderator of Cd^2+^ toxicity, which increases in time. An innovative method for the fertilization of soil did not play as important a role in the reclamation of polluted soil as straw did. The efficiency of the applied alleviating substances may be arranged as follows: barley straw > basalt meal > brown algae extract. Sowing spring barley improved the biochemical properties of the soil.
